# The influence of testosterone on the risk of cardiovascular events after percutaneous coronary intervention

**DOI:** 10.3389/fcvm.2022.998056

**Published:** 2022-12-22

**Authors:** Cheng-Hung Chiang, Wan-Ting Hung, En-Shao Liu, Tse-Hsuan Yang, Chin-Chang Cheng, Wei-Chun Huang, Guang-Yuan Mar, Feng-Yu Kuo

**Affiliations:** ^1^Cardiovascular Medical Center, Kaohsiung Veterans General Hospital, Kaohsiung, Taiwan; ^2^College of Medicine, National Yang Ming Chiao Tung University, Taipei, Taiwan; ^3^Department of Critical Care Medicine, Kaohsiung Veterans General Hospital, Kaohsiung, Taiwan; ^4^Department of Physical Therapy, Fooyin University, Kaohsiung, Taiwan; ^5^Graduate Institute of Clinical Medicine, Kaohsiung Medical University, Kaohsiung, Taiwan; ^6^Department of Pharmacy and Master Program, College of Pharmacy and Health Care, Tajen University, Pingtung, Taiwan

**Keywords:** testosterone, major adverse cardiovascular events, percutaneous coronary intervention, coronary artery disease, EPI—epidemiology

## Abstract

**Methods:**

Between 2015 and 2018, 580 men undergoing PCI at a tertiary referral hospital were divided into low (<3.25 ng/mL) and normal (≥3.25 ng/mL) testosterone groups. Major adverse cardiovascular event (MACE) was defined as the composite outcome of CV death, myocardial infarction, and target lesion revascularization/target vessel revascularization (TLR/TVR) during up to 48 months follow-up after PCI.

**Results:**

There were 111 and 469 patients in the low and normal testosterone groups, respectively, with the overall MACE rate of the former being higher than the latter (26.13% vs. 13.01%, *p* = 0.0006). Moreover, the overall TLR/TVR (20.72% vs. 11.73%, *p* = 0.0125) and myocardial infarction (3.6% vs. 0.85%, *p* = 0.0255) rates were significantly higher in those with low serum testosterone who also had a shorter average event-free survival analysis of MACE (25.22 ± 0.88 months) than those with normal testosterone levels (35.09 ± 0.47 months, log-rank *p* = 0.0004). Multiple logistic regression demonstrated an association between low serum testosterone (<3.25 ng/mL) and a higher MACE rate [odds ratio: 2.06, 95% confidence interval (CI) 1.21–3.51, *p* = 0.0081]. After adjusting for variables in a Cox regression model, hazard ratios (HRs) for MACE (HR: 1.88, 95% CI: 1.20–2.95, *p* = 0.0058) and TLR/TVR (HR: 1.73, 95% CI: 1.06–2.83, *p* = 0.0290) rates were higher in the low testosterone group than those in the normal testosterone group.

**Conclusion:**

Low serum testosterone concentrations were associated with a higher risk of MACE and TLR/TVR after PCI than those with normal testosterone levels.

## Introduction

Testosterone, which is synthesized mainly by the testes and to a smaller extent by the adrenal glands, is the major male sex hormone that has multiple important physiological functions. Not only it is essential for the maintenance of normal male reproductive function, but it also has a key role to play in the central nervous, musculoskeletal, and cardiovascular (CV) systems. Moreover, testosterone has been shown to be crucial to the regulation of carbohydrate, lipid, and protein metabolism as reflected in its positive effects on glucose control, muscle growth, and adipogenesis (1, 2). Men are well known to have an increased probability of developing coronary artery disease (CAD) together with a higher rate of mortality compared with women mainly attributable to the cardioprotective effect from estrogen and possible deleterious influence from testosterone on the CV system (3). However, the latter concern is being challenged (4). In fact, physiological concentration of testosterone tends to be cardioprotective in males (5). Indeed, testosterone, which is a potent vasodilator through calcium antagonistic action, has been reported to be protective against angina, especially in men with a low testosterone level (6, 7). Consistently, previous studies have shown an association between a low testosterone level and an increased risk of CV diseases (8–10) as well as a lower testosterone level in patients with CAD compared to those without (11, 12). Patients with a low testosterone level are more likely to have atherosclerotic plaques, endothelial dysfunction, and higher levels of high-sensitivity C-reactive protein (13).

Although percutaneous coronary intervention (PCI) can be life-saving and achieve long-term symptom relief (14), CV events after PCI are not uncommon in this patient population who usually has other accompanying physical conditions (15, 16). The current study aimed at elucidating the relationship between serum testosterone level and the risk of CV events in male patients after PCI that has not been adequately addressed in previous investigations.

## Materials and methods

### Study population

This is a retrospective cohort study focusing on male adult patients (defined as age over 20 years) receiving PCI at a single tertiary referral hospital between January 1, 2015 and December 31, 2018. Of the 5,416 patients undergoing PCI within the study period, 1,094 were excluded due to female gender or age below 20 years. In addition, 3,742 patients were excluded due to no measurement record of testosterone or loss of follow-up for more than 6 months. Finally, 580 patients were enrolled in this study (Supplementary Figure 1). The protocol and procedures of the current study were reviewed and approved by the institutional review board (IRB) of our institute. The IRB agreement number was VGHKS19-CT7-13.

### Study parameters

Baseline characteristics [i.e., age, body mass index (BMI), comorbidities, and smoking habit], results of laboratory studies on admission [i.e., glycated hemoglobin (HbA1c), estimated glomerular filtration rate (eGFR), total cholesterol, including high-density lipoprotein (HDL) and low-density lipoprotein (LDL), LDL 1-year after PCI, triglyceride (TG), and testosterone], and medications after PCI [i.e., anti-platelets, beta blockers, statins, angiotensin II receptor blockers (ARBs), and angiotensin-converting enzyme inhibitors (ACEIs)], and data on hospital admission and re-admission were collected. The cardiac (i.e., left ventricular ejection fraction) and PCI data [i.e., coronary artery disease status (single, double, or triple vessel disease), numbers of coronary artery treated, sites of coronary artery treated [left main, left anterior descending (LAD), proximal LAD, left circumflex, right, or ramus intermediate coronary artery], numbers of stent implanted, types of stent implantation [bare-metal stent or drug-eluting stent (DES)], types of coronary lesion [type A, B1, B2 or C according to modified American College of Cardiology/American Heart Association (ACC/AHA) stenosis morphology classification (17)], coronary artery calcification (18), SYNTAX score (19), rates of intravascular ultrasound or optical coherence tomography use, rates of bifurcation lesion, rates of chronic total occlusion lesion, and procedure success rates of PCI were also collected.

### Definitions

Major cardiovascular event (MACE) was defined as the composite outcome of CV death, myocardial infarction, and target lesion revascularization/target vessel revascularization (TLR/TVR) up to 48 months follow-up after PCI. Patients were further divided into low testosterone group (defined as serum testosterone level < 3.25 ng/mL) and normal testosterone group (defined as serum testosterone ≥ 3.25 ng/mL). The cutoff serum testosterone level to define hypogonadism remains controversial. The European Association of Urology (EAU) guidelines have defined a total testosterone threshold of 12.1 mmol/L (349 ng/dL) because this constitutes the lower end of the 2.5 percentile of population norms (20, 21). In contrast, the American Urology Association (AUA) sets a threshold of less than 10.4 mmol/L (300 ng/dL) for defining a low testosterone level (21, 22). To be more objective in data interpretation, our study used the mean of the thresholds defined by EAU and AUA (i.e., 3.25 ng/mL) as the cutoff level of serum testosterone to differentiate a normal from a low serum testosterone level in patients after PCI. A previous investigation into the associations of acute coronary syndrome with low testosterone levels and male sexual symptoms adopted a cutoff value of 3.2 ng/mL, which was similar to that of the present study (23).

### Statistical analyses

Continuous variables and categorical variables are shown as mean ± standard deviation and number with percentage (*n*, %), respectively. Independent sample *t*-tests and Chi-squared tests were performed to illustrate any difference in patient characteristics, laboratory readings, medications, and re-admission, by the testosterone groups. Kaplan–Meier survival analysis was performed to compare the survival probability of MACE between the low and normal testosterone groups. To identify significant risk factors, we used logistic regression to assess the association between characteristics and MACE expressed as odds ratio (OR) and 95% confidence interval (CI). Furthermore, we evaluated the hazard ratio (HR) and 95% CI between testosterone level and MACE outcomes with Cox proportional hazards regression, which can be used for survival-time (time-to-event) outcomes on one or more predictors.

The consumption of cox proportional hazards model is defined as the following:


$$l\,o\,g\,\left[\frac{h\,\left(t\,i\right)}{h_{0}\,\left(t\,i\right)}\right]=\beta_{1}\,\chi_{1}+\beta_{2}\,\chi_{2}+\beta_{3}\,\chi_{3}+\ldots +\beta_{n}\,\chi_{n}$$

where h_0_(ti) is the baseline hazard and h(ti) is the hazard function, which represent the probability of having the clinical event (ex: MACE, TVR/TLR, MI, or death) at time ti or the following endpoint. The terms X_1_, X_2_, X_3_ to X_*n*_ are covariates, which always been the independent impact factors, and β_1_, β_2_, to β_*n*_ are the corresponding regression coefficients (24). If the *p*-value is less than 0.05, then it is defined to be statistically significant. Statistical analysis was conducted by using software IBM SPSS 22.0 and SAS 9.4.

## Results

Of the 580 eligible patients, 111 were assigned to the low testosterone group (i.e., serum testosterone level < 3.25 ng/mL) and 469 fit the criterion for normal testosterone group (i.e., serum testosterone ≥ 3.25 ng/mL). The flowchart of enrollment is presented in Supplementary Figure 1. The baseline characteristics of all participants are listed in Supplementary Table 1. Patients in the low testosterone group had higher mean BMI (27.58 ± 4.34 vs. 26.12 ± 3.83, *p* = 0.0014), HbA1c (7.06 ± 1.43 vs. 6.73 ± 1.26, *p* = 0.0175), TG (158.23 ± 91.23 vs. 133.77 ± 95.91, *p* = 0.0151), and testosterone (2.48 ± 0.70 vs. 5.25 ± 3.25, *p* < 0.0001) as well as lower mean total cholesterol (148.29 ± 33.2 vs. 156.26 ± 34.54, *p* = 0.0280) and HDL (39.72 ± 8.62 vs. 44.34 ± 10.48, *p* < 0.0001) concentrations compared to those in the normal testosterone group. Regarding comorbidities, patients in the low testosterone group had a higher prevalence of hypertension (78.38% vs. 67.80%, *p* = 0.0291), diabetes mellitus (50.45% vs. 39.66%, *p* = 0.0381), and uremia (9.01% vs. 2.77%, *p* = 0.0025) compared to those with a normal testosterone level. In respect of medications, patients in the low testosterone group had a lower rate of dual-antiplatelet therapy for more than 6 months (57.66% vs. 72.49%, *p* = 0.0022) after PCI than those in the normal testosterone group. The cardiac and PCI data of all participants are presented in Supplementary Table 2. Male patients with low serum testosterone had a lower rate of LAD coronary artery treated than those with normal serum testosterone (55.86% vs. 66.95%, *p* = 0.0278). However, the rate of proximal LAD treated did not differ significantly (26.13% vs. 33.69%, *p* = 0.1253). Otherwise, all the other characteristics had no significant difference between male patients with low and normal serum testosterone.

The CV events of the two groups are summarized in Supplementary Table 3. The overall MACE rate of all the patients was 15.52%. It was higher in the low testosterone group than that in the normal testosterone group (26.13% vs. 13.01%, respectively, *p* = 0.0006). Regarding the need for revascularization, the overall TLR/TVR rate was 13.45%. Similarly, it was higher in the low testosterone group compared to that in the normal testosterone group (20.72% vs. 11.73%, respectively, *p* = 0.0125). In addition, the myocardial infarction rate was higher in the low testosterone group than that in the normal testosterone group (3.6% vs. 0.85%, respectively, *p* = 0.0255), giving an overall rate of 1.38%. The all-cause mortality rate of the low testosterone group compared to those with normal testosterone showed 1.8% vs. 0.43% (*p* = 0.1154).

**FIGURE 1:**
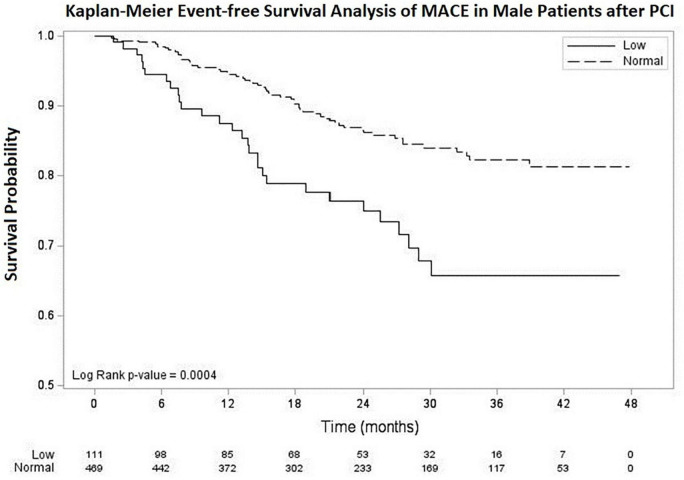
Kaplan–Meier survival curve for MACE in male patients after PCI with low and normal testosterone level. The average survival time was 25.22 ± 0.88 months in low testosterone group (testosterone < 3.25 ng/mL) and 35.09 ± 0.47 months in normal testosterone group (testosterone ≥ 3.25 ng/mL). MACE, major adverse cardiovascular events. PCI, percutaneous coronary intervention.

Kaplan–Meier event-free survival analysis of MACE up to 48 months of follow-up is shown in Figure 1. The overall mean follow-up period was 24.3 ± 12.7 months [Median (minimum, maximum): 23.7 (0.6, 47.8) months], 22.3 ± 12.3 months in low testosterone group [Median (minimum, maximum): 23.4 (1.0, 46.9) months], and 24.7 ± 12.8 months [Median (minimum, maximum) 23.9 (0.6, 47.8) months] in normal testosterone group. The mean event-free survival time for MACE was 25.22 ± 0.88 months in the low testosterone group and 35.09 ± 0.47 months in the normal testosterone group. The log-rank *p*-value was 0.0004.

**FIGURE 2:**
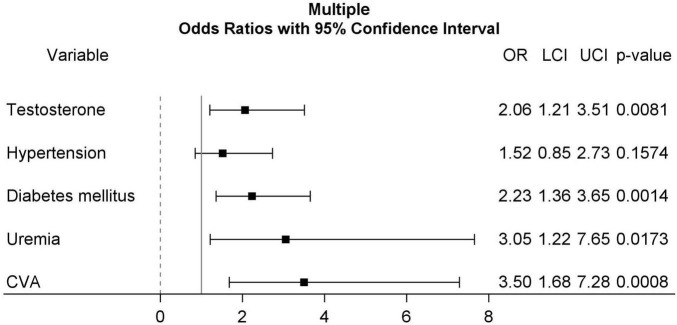
Forest plots for multiple logistic regression including testosterone (≥3.25 ng/mL, as reference group), hypertension, diabetes mellitus, uremia, and CVA. CVA, cerebrovascular accident.

The association between patient characteristics and MACE by univariable and multiple logistic regression is demonstrated in Table 1. In univariable logistic regression, patients with serum testosterone < 3.25 ng/mL (OR: 2.37, 95% CI: 1.42–3.91, *p* = 0.0008), hypertension (OR: 2.04, 95% CI: 1.17–3.58, *p* = 0.0125), diabetes mellitus (OR: 2.85, 95% CI: 1.79–4.54, *p* < 0.0001), uremia (OR: 5.55, 95% CI: 2.37–13.01, *p* < 0.0001), and cerebrovascular accident (CVA) (OR: 3.88, 95% CI: 1.95–7.74, *p* = 0.0001) were associated with higher MACE rates. Multiple logistic regression identified serum testosterone < 3.25 ng/mL (OR: 2.06, 95% CI: 1.21–3.51, *p* = 0.0081), diabetes mellitus (OR: 2.23, 95% CI: 1.36–3.65, *p* = 0.0014), uremia (OR: 3.05, 95% CI: 1.22–7.65, *p* = 0.0173), and CVA (OR: 3.50, 95% CI: 1.68–7.28, *p* = 0.0008) as significant predictors of higher MACE rates. Forest plot for multiple logistic regression is shown in Figure 2.

**TABLE 1:** Association between the characteristics of patients and major adverse cardiovascular event by univariable and multiple logistic regression.

	Univariable			Multiple*		
	OR	(95% CI)	*P-*value	OR	(95% CI)	*P*-value
Age	1.00	(0.98, 1.02)	0.9518	–	–	–
BMI	1.00	(0.94, 1.06)	0.9766	–	–	–
LDL	0.99	(0.99, 1.00)	0.0772	–	–	–
LDL 1-year after PCI	0.99	(0.99, 1.00)	0.1969	–	–	–
Testosterone**	2.37	(1.42, 3.91)	0.0008	2.06	(1.21, 3.51)	0.0081
Hypertension	2.04	(1.17, 3.58)	0.0125	1.52	(0.85, 2.73)	0.1574
Diabetes mellitus	2.85	(1.79, 4.54)	<0.0001	2.23	(1.36, 3.65)	0.0014
Uremia	5.55	(2.37, 13.01)	<0.0001	3.05	(1.22, 7.65)	0.0173
Heart failure	1.26	(0.75, 2.10)	0.3836	–	–	–
Hypercholesterolemia	1.31	(0.83, 2.07)	0.2391	–	–	–
Current smoker	0.64	(0.37, 1.12)	0.1190	–	–	–
Ex-smoker	1.21	(0.59, 2.49)	0.6113	–	–	–
COPD	0.91	(0.20, 4.12)	0.8975	–	–	–
Previous MI	1.39	(0.88, 2.17)	0.1575	–	–	–
CVA	3.88	(1.95, 7.74)	0.0001	3.50	(1.68, 7.28)	0.0008
ACS during admission	1.34	(0.84, 2.12)	0.2170	–	–	–
LAD treated	0.93	(0.58, 1.48)	0.7482	–	–	–
DAPT (>6 month)	0.67	(0.42, 1.07)	0.0964	–	–	–
DAPT (>12 month)	0.67	(0.40, 1.13)	0.1353	–	–	–
Beta blocker	1.14	(0.71, 1.82)	0.5909	–	–	–
Statin	1.14	(0.72, 1.78)	0.5789	–	–	–
ACEI/ARB	1.25	(0.79, 1.99)	0.3393	–	–	–

*Multiple regression variables were including testosterone, hypertension, diabetes mellitus, uremia, and CVA, which according to the results of univariable logistic regression.

**Testosterone ≥ 3.25 ng/mL as reference groups.

OR, odds ratio; BMI, body mass index; COPD, chronic obstructive pulmonary disease; MI, myocardial infarction; CVA, cerebral vascular accident; ACS, acute coronary syndrome; LAD, left anterior descending; DAPT, dual anti-platelet therapy; ARB, angiotensin II receptor blocker; ACEI, angiotensin-converting enzyme inhibitor.

The HR for CV events based on testosterone level are summarized in Table 2. Before adjustment with patients having a serum testosterone concentration ≥ 3.25 ng/mL serving as the reference group, the rates of MACE (HR: 2.19, 95% CI: 1.41–3.41, *p* = 0.0005), TVR/TLR (HR: 1.93, 95% CI: 1.19–3.14, *p* = 0.0082), and myocardial infarction (HR: 4.54, 95% CI: 1.13–18.15, *p* = 0.0325) were higher in patients with serum low testosterone level compared to those with normal testosterone level. After adjusting for diabetes mellitus, uremia, and CVA, with patients having a normal serum testosterone level serving as the reference group, the rates of MACE (HR: 1.88, 95% CI: 1.20–2.95, *p* = 0.0058) and TLR/TVR (HR: 1.73, 95% CI: 1.06–2.83, *p* = 0.0290) remained higher in the low testosterone group than those in the normal testosterone group.

**TABLE 2:** Hazard ratios (HR) and 95% confidence interval (95% CI) for cardiovascular events according to testosterone level (testosterone ≥ 3.25 ng/mL as reference group) by cox regression.

	HR	95% CI	*P*-value
**Unadjusted**			
MACE	2.19	(1.41, 3.41)	0.0005
TVR/TLR	1.93	(1.19, 3.14)	0.0082
MI	4.54	(1.13, 18.15)	0.0325
Death	4.54	(0.64, 32.26)	0.1303
**Adjusted** *			
MACE	1.88	(1.20, 2.95)	0.0058
TVR/TLR	1.73	(1.06, 2.83)	0.0290
MI	2.27	(0.51, 10.12)	0.2794
Death	4.01	(0.56, 28.56)	0.1656

*Adjusted variables were diabetes mellitus, uremia, and CVA, which according to the results of multiple logistic regression.

MACE, major adverse cardiac event; TVR/TLR, target vessel revascularization/target lesion revascularization; MI, myocardial infarction.

## Discussion

Coronary artery disease is associated with a low serum testosterone level and about 20% of men with CAD have testosterone levels compatible with hypogonadism in the previous study (25). The severity of CAD has also been shown to correlate with the degree of testosterone deficiency (26). However, previous epidemiology study failed to found the associated between testosterone and atherosclerosis (27). We investigated CAD patients after receiving PCI and would like to evaluate the influence of testosterone in this patient group. Our study showed that 19.13% (111/580) of male patients after PCI had a serum testosterone level below 3.25 ng/mL (defined as low serum testosterone) and the ratio was similar to the previous study. About the severity of CAD shown in Supplementary Table 2, including CAD status (single, double, or triple vessel disease) and types of coronary lesion (type A, B1, B2, or C), coronary artery calcification (mild, moderate, or severe), and SYNTAX score, we found that there was no significant difference between low and normal testosterone group, compatible to the finding of previous study (27). The patients enrolled in our study group were those who already had positive finding in non-invasive stress test or computerize tomography coronary angiogram, who referred from other hospital for the complex PCI, or who suffered from acute coronary syndrome. In this patient group, the complexity of CAD was similar. This may be the reason why our data did not have significant difference in CAD severity between low and normal testosterone groups. In addition, the similarity of coronary artery severity and complexity between low and normal testosterone groups could minimize the difference of PCI influence on MACE in our study.

Testosterone is a key regulator in the maintenance of metabolic homeostasis and testosterone deficiency is frequently found in men with metabolic syndrome. It appears that hypogonadism predisposes men to endothelium dysfunction, inflammation, insulin resistance, obesity, abnormal lipid profiles, and borderline or overt hypertension (28). Previous studies have consistently demonstrated that metabolic syndrome is more prevalent in men with low concentrations of testosterone (29). One meta-analysis has demonstrated that having metabolic syndrome independently predicts low testosterone level (30). In our study, patients after PCI with lower serum testosterone were associated higher prevalence of signs for metabolic syndrome, including higher BMI, HbA1c, and TG, lower HDL, and more hypertension and diabetes mellitus, and the results were identical to previous studies. Metabolic syndrome in male patients with hypogonadism may be one of the contributors to higher risks of MACE after PCI than those with normal testosterone level.

Studies discussed about the influence of testosterone on long-term event rates after PCI or acute coronary syndrome are scarce. One study by Chmiel et al. focused on low testosterone and sexual symptoms in men with acute coronary syndrome. During 18.3 months of follow-up, Kaplan–Meier analysis revealed that patients with low testosterone and sexual symptoms had a higher risk of having a MACE compared to those without low testosterone and sexual symptoms with log-rank test HR 2.6 (95% CI: 1.2–5.3; *p* < 0.01) (23). Another recent study by Gencer et al. evaluated total testosterone level in patients with acute coronary syndrome. During 1-year of follow-up, patients in the lowest testosterone tertile had a mortality rate of 5.4% compared with 2.9% in the highest tertile, with an adjusted HR of 1.26 (95% CI: 0.57–2.78; *p* = 0.565) (31). To our knowledge, the present investigation is the first observational study to elucidate the influence of testosterone in male patients after PCI on their clinical outcomes. The strength of our study is that the duration of follow-up after PCI was long, up to 4 years. Our data demonstrated an association between low serum testosterone level and high MACE, as compared to those with normal serum testosterone level. Multiple logistic regression revealed a higher OR of MACE (OR: 2.06, 95% CI: 1.21–3.51, *p* = 0.0081) and Cox regression demonstrated higher adjusted HR ratios for MACE (HR: 1.88, 95% CI: 1.20–2.95, *p* = 0.0058) in those with a low testosterone level. Kaplan–Meier event-free analysis of MACE revealed a shortened event-free time (25.22 ± 0.88 vs. 35.09 ± 0.47 months, log-rank *p* = 0.0004) in patients with a low serum testosterone level.

A low testosterone level has been found to be associated with an increased risk of mortality (32, 33). A meta-analysis by Araujo et al. included 12 studies involving more than 17,000 participants revealed that low endogenous testosterone levels were associated with both overall and CV mortality. However, there was considerable heterogeneity in these studies resulting from study and subject characteristics (8). In our study, we enrolled patients only with regular follow-up after PCI and the mortality rate during follow-up period was 1.8% in low testosterone group and 0.43% in normal testosterone group. In both groups, the mortality rate was low, and these may be the reason for that there was no significant difference in mortality between low and normal testosterone groups (*p* = 0.1154).

There were several limitations in our study. First, not all patients had available serum testosterone data for analysis because of the retrospective nature of the current cohort study. During the follow-up period, only 580 out of 4,322 potentially eligible male patients (13%) provided serum testosterone data without a loss of follow-up. Second, significant differences in some of the characteristics between the low and normal testosterone groups such as BMI, HbA1c, TG, total cholesterol, HDL, and LDL may influence the MACE rate. Third, the severity and complexity of coronary artery during PCI may have impacts on the CV events and revascularization during the follow-up period. To evaluate these influences, we compared most of the characteristics about PCI, which are listed in Supplementary Table 2, and the results revealed that only the proportion of LAD treatment had the significant difference. We believe that the similarity of coronary artery severity and complexity between low and normal testosterone groups could minimize the difference of PCI influence on MACE in this study. Fourth, previous study has shown an age-related decline in serum testosterone and approximately 25% of men over 65 years of age had low serum testosterone (34). In this study, we did not evaluate the impact of age on MACE in patients after PCI. However, the mean age of low and normal serum testosterone groups did not have significant difference. This may minimize the impact of age influence on MACE in different testosterone concentration groups. Fifth, the low complexity of the CAD in our study population, as confirmed by SYNTAX score, suggests a low generalization of the current results to other patients affected by more complex CAD.

## Conclusion

Our study revealed a higher risk of MACE among male patients after PCI with a low testosterone level than those with a normal testosterone level after full adjustment. Due to the retrospective nature of this study, these results should be interpreted as hypothesis generating and they should be confirmed by randomized trial.

## Data availability statement

The raw data supporting the conclusions of this article will be made available by the authors, without undue reservation.

## Ethics statement

This study was reviewed and approved by the institutional review board of the KSVGH that waived the requirement for an informed consent from the patients in accordance with the relevant guidelines and regulations.

## Author contributions

All authors involved in drafting the article or revising it, took responsibility for all aspects of the reliability and freedom from bias of the data presented and their discussed interpretation, and approved the final version to be published.

## Supplementary material

Supplementary Figure 1 The flowchart of the study design. CAD, coronary artery disease; PCI, percutaneous coronary intervention; BMI, body mass index; HbA1c, glycated hemoglobin; eGFR, estimated glomerular filtration rate; HDL, high-density lipoprotein; LDL, low-density lipoprotein; LVEF, left ventricular ejection fraction; IVUS/OCT, intravascular ultrasound/optical coherence tomography; ACC/AHA, the American College of Cardiology/the American Heart Association; CAC, coronary artery calcification; CTO, chronic total occlusion; ARB, angiotensin II receptor blocker; ACEI, angiotensin-converting enzyme inhibitor.
