# Supplementary material for: The influence of testosterone on the risk of cardiovascular events after percutaneous coronary intervention
Source: Front Cardiovasc Med. 2022 Dec 22;9:998056. doi: 10.3389/fcvm.2022.998056 (PMC9815835; doi:10.3389/fcvm.2022.998056)
Supplement: Supplementary file 1 [file Data_Sheet_1.docx]

**Supplementary Table 1.** The baseline characteristics of patients with different level of testosterone concentration.

|  | Overall | Low Testosterone (T) | Normal Testosterone (T) | *p*-value |
| --- | --- | --- | --- | --- |
|  |  | T < 3.25 ng/mL | T ≥ 3.25 ng/mL |  |
|  | N=580 | N=111 | N=469 |  |
| Characteristics, mean±SD |  |  |  |  |
| Age (years) | 61.98±11.66 | 61.17±13.14 | 62.17±11.29 | 0.4608 |
| BMI (kg/m^2^) | 26.40±3.97 | 27.58±4.34 | 26.12±3.83 | 0.0014 |
| HbA1c (%) | 6.79±1.30 | 7.06±1.43 | 6.73±1.26 | 0.0175 |
| eGFR (ml/min/1.73) | 64.19±21.96 | 61.57±27.45 | 64.81±20.43 | 0.2444 |
| Triglyceride (mg/dL) | 138.48±95.44 | 158.23±91.23 | 133.77±95.91 | 0.0151 |
| Total Cholesterol (mg/dL) | 154.74±34.40 | 148.29±33.2 | 156.26±34.54 | 0.0280 |
| HDL (mg/dL) | 43.45±10.30 | 39.72±8.62 | 44.34±10.48 | <0.0001 |
| LDL (mg/dL) | 87.78±29.22 | 83.40±27.99 | 88.82±29.43 | 0.0785 |
| LDL one-year after PCI (mg/dL) | 84.38±30.03 | 79.62±28.82 | 85.47±30.22 | 0.0867 |
| Testosterone (ng/mL) | 4.72±1.88 | 2.48±0.70 | 5.25±3.25 | <0.0001 |
| Previous medical history, n (%) |  |  |  |  |
| Hypertension | 405 (69.83) | 87 (78.38) | 318 (67.80) | 0.0291 |
| Diabetes mellitus | 242 (41.72) | 56 (50.45) | 186 (39.66) | 0.0381 |
| Uremia | 23 (3.97) | 10 (9.01) | 13 (2.77) | 0.0025 |
| Heart failure | 134 (23.10) | 23 (20.72) | 111 (23.67) | 0.5078 |
| Hypercholesterolemia | 302 (52.07) | 51 (45.95) | 251 (53.52) | 0.1510 |
| COPD | 14 (2.41) | 1 (0.9) | 13 (2.77) | 0.2481 |
| Previous MI | 276 (47.59) | 52 (46.85) | 224 (47.76) | 0.8623 |
| Previous CABG | 19 (3.28) | 2 (1.80) | 17 (3.62) | 0.3319 |
| CVA | 39 (6.72) | 7 (6.31) | 32 (6.82) | 0.8450 |
| ACS during admission | 199 (34.31) | 35 (31.53) | 164 (34.97) | 0.4928 |
| Current smoker | 155 (26.72) | 24 (21.62) | 131 (27.93) | 0.1767 |
| Ex-smoker | 56 (9.66) | 10 (9.01) | 46 (9.81) | 0.7977 |
| Medication, n (%) |  |  |  |  |
| Aspirin | 496 (85.52) | 91 (81.98) | 405 (86.35) | 0.2392 |
| Clopidogrel | 352 (60.69) | 65 (58.56) | 287 (61.19) | 0.6092 |
| Ticagrelor | 173 (29.83) | 34 (30.63) | 139 (29.64) | 0.8371 |
| Ticlopidine | 2 (0.34) | 0 (0.00) | 2 (0.43) | 0.4907 |
| DAPT (>6 month) | 404 (69.66) | 64 (57.66) | 340 (72.49) | 0.0022 |
| DAPT (>12 month) | 174 (30.00) | 30 (27.03) | 144 (30.7) | 0.4472 |
| Beta blocker | 192 (33.10) | 44 (39.64) | 148 (31.56) | 0.1037 |
| Statin | 268 (46.21) | 48 (43.24) | 220 (46.91) | 0.4861 |
| ACEI/ARB | 200 (34.48) | 45 (40.54) | 155 (33.05) | 0.1354 |

BMI, body mass index. HbA1c, glycated hemoglobin. eGFR, estimated glomerular filtration rate. HDL, high-density lipoprotein. LDL, low-density lipoprotein. PCI, percutaneous coronary intervention. COPD, chronic obstructive pulmonary disease. MI, myocardial infarction. CABG, coronary artery bypass graft. CVA, cerebrovascular accident ACS, acute coronary syndrome. DAPT, dual antiplatelet therapy. ACEI, angiotensin converting enzyme inhibitor. ARB, angiotensin II receptor blocker.

**Supplementary Table 2.** The cardiac and percutaneous coronary intervention data of patients with different level of testosterone concentration.

|  | Overall | Low Testosterone (T) | Normal Testosterone (T) | *p*-value |
| --- | --- | --- | --- | --- |
|  |  | T < 3.25 ng/mL | T ≥ 3.25 ng/mL |  |
|  | N=580 | N=111 | N=469 |  |
| Characteristics, mean±SD |  |  |  |  |
| LVEF (%) | 55.61±8.00 | 55.89±8.01 | 55.54±8.00 | 0.6873 |
| SYNTAX score | 15.17±7.12 | 14.29±7.15 | 15.38±7.11 | 0.1478 |
| Coronary artery treated (n) | 1.63±0.74 | 1.60±0.72 | 1.64±0.74 | 0.6659 |
| Stent implantation (n) | 1.88±0.99 | 1.87±0.95 | 1.88±1.00 | 0.9220 |
| CAD (n, %) |  |  |  | 0.7283 |
| SVD | 147 (25.48) | 25 (22.52) | 122 (26.18) |  |
| DVD | 171 (29.64) | 34 (30.63) | 137 (29.40) |  |
| TVD | 259 (44.89) | 52 (46.85) | 207 (44.42) |  |
| Lesion location, n (%) |  |  |  |  |
| LM | 69 (11.96) | 13 (11.71) | 56 (12.02) | 0.9290 |
| LAD | 374 (64.82) | 62 (55.86) | 312 (66.95) | 0.0278 |
| Proximal LAD | 187 (32.24) | 29 (26.13) | 158 (33.69) | 0.1253 |
| LCx | 213 (36.92) | 42 (37.84) | 171 (36.70) | 0.8226 |
| RCA | 278 (48.18) | 60 (54.05) | 218 (46.78) | 0.1682 |
| Ramus intermediate | 7 (1.21) | 1 (0.90) | 6 (1.29) | 0.7381 |
| Type of stent, n (%) |  |  |  |  |
| BMS | 68 (11.72) | 14 (12.61) | 54 (11.51) | 0.7463 |
| DES | 531 (91.55) | 101 (90.99) | 430 (91.68) | 0.8133 |
| IVUS/OCT, n (%) | 194 (33.45) | 35 (31.53) | 159 (33.90) | 0.6431 |
| Modified ACC/AHA lesion type, n (%) |  |  |  | 0.4863 |
| A | 186 (32.07) | 37 (33.33) | 149 (31.77) |  |
| B1 | 78 (13.45) | 13 (11.71) | 65 (13.86) |  |
| B2 | 102 (17.59) | 15 (13.51) | 87 (18.55) |  |
| C | 214 (36.90) | 46 (41.44) | 168 (35.82) |  |
| Coronary artery calcification, n (%) |  |  |  | 0.6085 |
| None/Mild | 332 (57.24) | 59 (53.15) | 273 (58.21) |  |
| Moderate | 156 (26.90) | 32 (28.83) | 124 (26.44) |  |
| Severe | 92 (15.86) | 20 (18.02) | 72 (15.35) |  |
| Bifurcation, n (%) | 250 (43.10) | 49 (44.14) | 201 (42.86) | 0.8055 |
| CTO, n (%) | 99 (17.07) | 19 (17.12) | 80 (17.06) | 0.9880 |
| Procedure success, n (%) | 564 (97.24) | 107 (96.40) | 457 (97.44) | 0.5455 |

LVEF, left ventricular ejection fraction. CAD, coronary artery disease. SVD, single vessel disease. DVD, double vessel disease. TVD, triple vessel disease. LM, left main. LAD, left anterior descending. LCx, left circumflex. RCA, right coronary artery. BMS, bare metal stent. DES, drug eluting stent. IVUS, intravascular ultrasound. OCT, optical coherence tomography. CTO, chronic total occlusion.

**Supplementary Table 3.** Cardiovascular events with different levels of testosterone

|  | Overall | Low Testosterone | Normal Testosterone | *p*-value |
| --- | --- | --- | --- | --- |
|  |  | T < 3.25 ng/mL | T ≥ 3.25 ng/mL |  |
|  | N=580 | N=111 | N=469 |  |
| Event, n (%) |  |  |  |  |
| MACE | 90 (15.52) | 29 (26.13) | 61 (13.01) | 0.0006 |
| TLR/TVR | 78 (13.45) | 23 (20.72) | 55 (11.73) | 0.0125 |
| MI | 8 (1.38) | 4 (3.60) | 4 (0.85) | 0.0255 |
| Death | 4 (0.69) | 2 (1.80) | 2 (0.43) | 0.1154 |

MACE, major adverse cardiac event. TLR/TVR, target lesion revascularization/target vessel revascularization. MI, myocardial infarction.
