# Supplementary figures and images for: The influence of testosterone on the risk of cardiovascular events after percutaneous coronary intervention
Source: Front Cardiovasc Med. 2022 Dec 22;9:998056. doi: 10.3389/fcvm.2022.998056 (PMC9815835; doi:10.3389/fcvm.2022.998056)

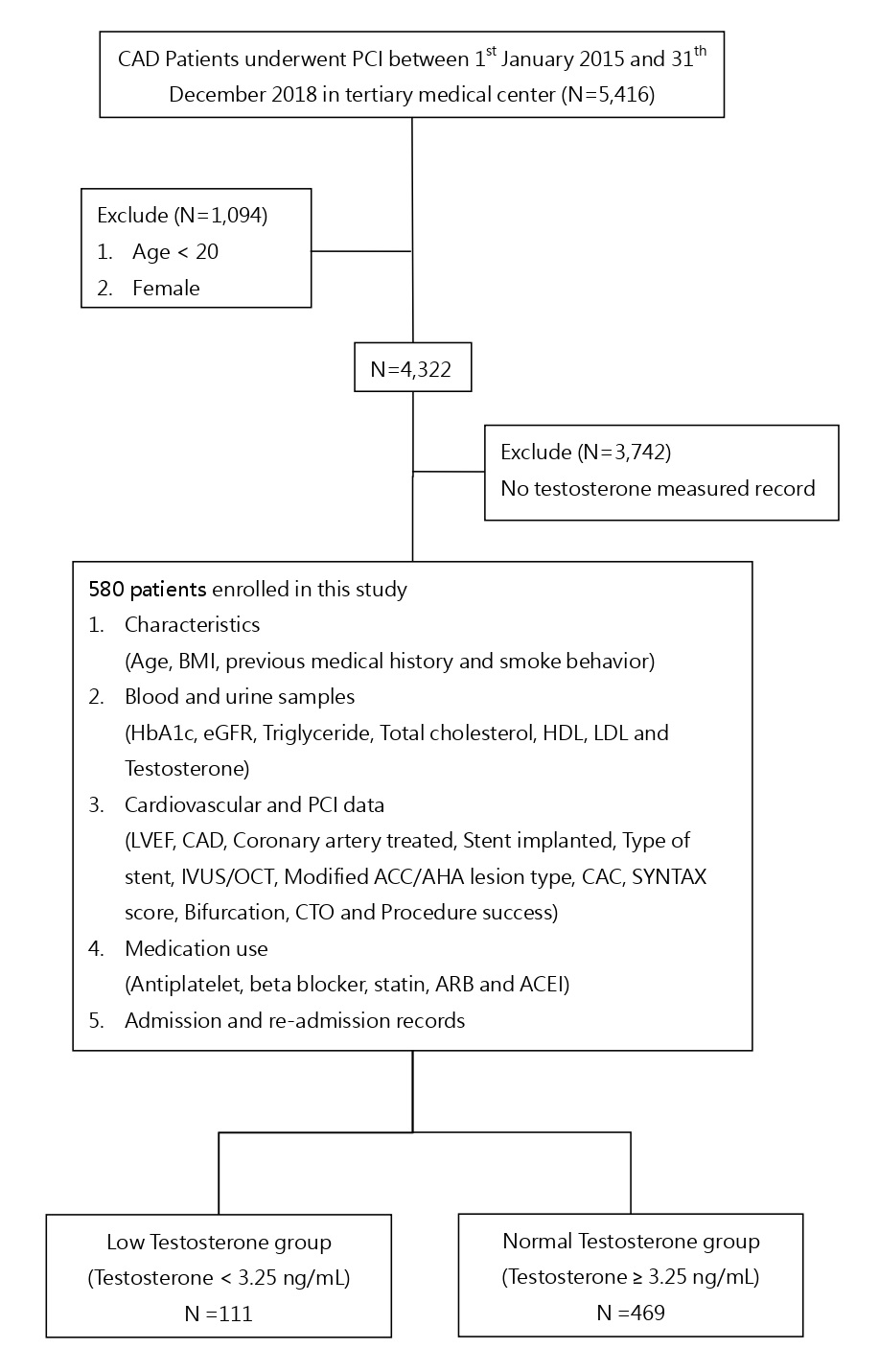

Supplement: Supplementary Figure 1 — The flowchart of the study design. CAD, coronary artery disease; PCI, percutaneous coronary intervention; BMI, body mass index; HbA1c, glycated hemoglobin; eGFR, estimated glomerular filtration rate; HDL, high-density lipoprotein; LDL, low-density lipoprotein; LVEF, left ventricular ejection fraction; IVUS/OCT, intravascular ultrasound/optical coherence tomography; ACC/AHA, the American College of Cardiology/the American Heart Association; CAC, coronary artery calcification; CTO, chronic total occlusion; ARB, angiotensin II receptor blocker; ACEI, angiotensin-converting enzyme inhibitor. [file Image_1.JPEG]
